# Association of *Strongyloides stercoralis* infection and type 2 diabetes mellitus in northeastern Thailand: Impact on diabetic complication-related renal biochemical parameters

**DOI:** 10.1371/journal.pone.0269080

**Published:** 2022-05-31

**Authors:** Manachai Yingklang, Apisit Chaidee, Rungtiwa Dangtakot, Chanakan Jantawong, Ornuma Haonon, Chutima Sitthirach, Nguyen Thi Hai, Ubon Cha’on, Sirirat Anutrakulchai, Supot Kamsa-ard, Somchai Pinlaor

**Affiliations:** 1 Department of Epidemiology and Biostatistics, Faculty of Public Health, Khon Kaen University, Khon Kaen, Thailand; 2 Chronic Kidney Disease Prevention in The Northeastern Thailand, Faculty of Medicine, Khon Kaen University, Khon Kaen, Thailand; 3 Department of Parasitology, Faculty of Medicine, Khon Kaen University, Khon Kaen, Thailand; 4 Department of Medical Technology, Faculty of Allied Health Science, Nakhon Ratchasima College, Nakhon Ratchasima, Thailand; 5 Department of Parasitology, Thai Nguyen University of Medicine and Pharmacy, Thai Nguyen, Vietnam; 6 Department of Biochemistry, Faculty of Medicine, Khon Kaen University, Khon Kaen, Thailand; 7 Department of Internal Medicine, Faculty of Medicine, Khon Kaen University, Khon Kaen, Thailand; Iran University of Medical Sciences, ISLAMIC REPUBLIC OF IRAN

## Abstract

**Background:**

Several studies have demonstrated that helminth infections provide a degree of protection against Type 2 diabetes mellitus (T2DM). However, the relationship between *Strongyloides stercoralis* infection and T2DM has scarcely been investigated and the protective effect of infection against development of diabetic complications is unclear. In this study, we aimed to investigate the relationship between *S*. *stercoralis* infection and T2DM in a rural area of Khon Kaen Province, Thailand. The impact of *S*. *stercoralis* infection on diabetic complication-related kidney function biochemical parameters and body-mass index (BMI) was also assessed.

**Methodology:**

Using a cross-sectional study design, *S*. *stercoralis* infection and T2DM assessments were conducted between October 2020 and May 2021. Associations between *S*. *stercoralis* infection, T2DM, and socioeconomic factors were analyzed using multivariable logistic regression analyses. Diabetic complication-related biochemical parameters relating largely to kidney function (estimated glomerular filtration rate (eGFR), urine albumin-to-creatinine ratio (UACR), serum creatinine, uric acid, alanine transaminase (ALT), and low-density lipoprotein cholesterol (LDL-C)) and BMI of participants with and without T2DM were compared between groups with or without *S*. *stercoralis* infection.

**Results:**

One hundred and seven out of 704 individuals (15.20%) were positive for *S*. *stercoralis*, and 283 people were diagnosed with T2DM. Of those with T2DM, 11.31% (32/283) were infected with *S*. *stercoralis* and of those without T2DM, 17.82% (75/421) were infected with *S*. *stercoralis*. Multivariate analysis revealed that T2DM was inversely correlated with *S*. *stercoralis* infection (Adjusted OR  =  0.49; 95% CI: 0.30, 0.78; *p*  = 0.003), while male, increasing age, lower education level, and alcohol intake were positively associated with infection. Those infected with *S*. *stercoralis* had lower eGFR levels and higher ALT and UACR levels than those in the uninfected group.

**Conclusion:**

This finding indicates that *S*. *stercoralis* infection was inversely associated with T2DM in northeastern Thailand, but participants infected with *S*. *stercoralis* had lower eGFR levels and higher ALT and UACR levels. Infection with *S*. *stercoralis* might lead to worse complication-related renal biochemical parameters.

## Introduction

Type 2 diabetes mellitus (T2DM) is defined by persistent hyperglycemia, chronic low-grade Th1-predominant systemic inflammation, and intestinal microbial dysbiosis [1]. The incidence of T2DM is increasing worldwide in various middle- and low-income countries, including Thailand [2]. T2DM is an established risk factor for developing complications in various organs, which are classified according to pathogenesis as macrovascular complications (including coronary-artery and peripheral-vascular disease) and microvascular complications (including renal, retinal, and possibly neuropathic disease) [3]. Co-morbidity of T2DM with other pathogens, including bacteria, viruses, fungi and parasites also has serious consequences [4, 5].

Several prior epidemiological studies have indicated an inverse association between prevalence of helminth infections (especially those caused by *Schistosoma* spp, soil-transmitted helminths and filarial nematodes) and incidence of T2DM in humans [6–9]. This might indicate that helminths exert a protective effect against T2DM. Increasing experimental evidence has revealed that helminth infection may help the host to improve insulin sensitivity and metabolic function against T2DM development by triggering innate and acquired immunoregulatory responses as well as by altering the gut microbiota [10–12]. These immune modulations are necessary for long-lived helminths species to dodge host attack [13].

In tropical and sub-tropical countries, strongyloidiasis is a neglected tropical disease caused by infection with the soil-transmitted helminth *Strongyloides stercoralis*. More than six hundred million people are infected with this intestinal helminth [14]. *Strongyloides stercoralis* has a complicated life cycle which includes parasitic, free-living, and autoinfection cycles [15]. Humans are infected with *S*. *stercoralis* infective filariform larvae (iL3) through skin penetration when in contact with soil, especially if shoes are not worn. *Strongyloides stercoralis* can persist in human hosts for several decades, the infection being perpetuated by the autoinfection cycle. Chronic strongyloidiasis is most often symptom free, while can be fatal in immunocompromised and organ transplantation patients due to hyperinfection: the helminth larvae can re-infect their host and disseminate to various organs [16, 17].

Previous studies have demonstrated the protective effect of helminth infections, including *S*. *stercoralis*, against T2DM, particularly noting alteration of systemic cytokines, chemokines, adipokines and biochemical parameters that favor protection from insulin resistance [18–20]. Recent study has indicated that infection with *S*. *stercoralis* could play a beneficial role by limiting T2DM-related vascular complications [21]. However, the mechanism underlying the relationship between helminth infection, particularly *S*. *stercoralis*, and T2DM is largely unclear. Community-based studies of this association also remain few. T2DM is a known risk factor for developing complications in various organs. However, the protective effect of *S*. *stercoralis* infection against multi-morbidity complications, especially kidney disease, is unknown—hence, further study is required.

In this study, we aimed to investigate the association between *S*. *stercoralis* infection and T2DM in Khon Kaen Province, Thailand, where a high prevalence of infection has been reported [22–24]. We also extended the analysis to assess the interaction between *S*. *stercoralis* infection and biochemical parameters related to kidney function, and also body-mass index (BMI) in participants with and without T2DM.

## Materials and methods

### Human ethical statement

This study was approved by the human ethical review committee of Khon Kaen University, Thailand (HE631573) following the principles of the Declaration of Helsinki. Before stool, blood and urine samples were collected, participants were required to complete and sign the written informed-consent forms. At the end of the study, participants infected with *S*. *stercoralis* were treated with a single dose of ivermectin (200 μg/kg body weight), while other intestinal parasitic infections were treated with albendazole, praziquantel or metronidazole as appropriate.

### Study population and study design

**Fig 1:**
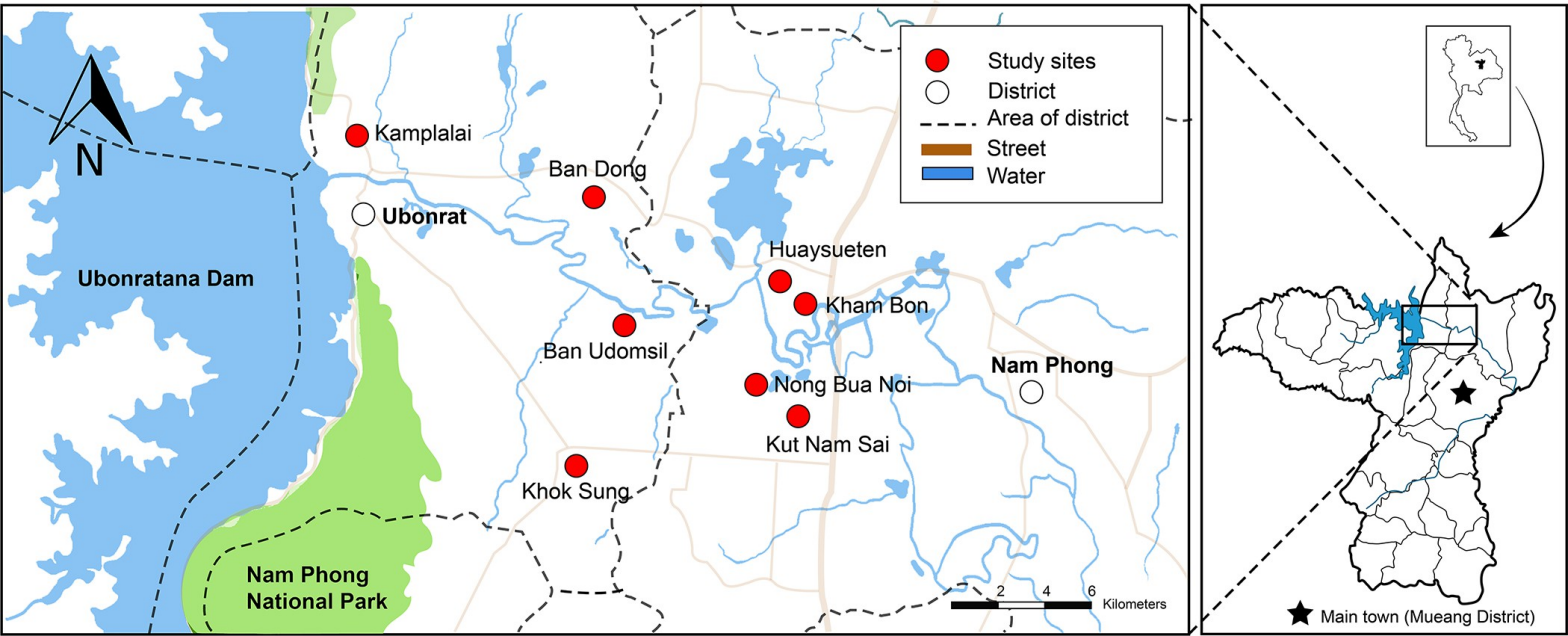
Geographical area of this study in Khon Kaen Province, northeastern Thailand. Map obtained from USGS National Map Viewer (public domain): http://viewer.nationalmap.gov/viewer/. The base map is visualized based on the OpenStreetMap elements tags. Reprinted from OpenStreetMap under a CC BY license, with permission from OpenStreetMap, original copyright 2020. Base map ©OpenStreetMap contributors. All other layers were produced by the authors and are copyright-free.

**Fig 2:**
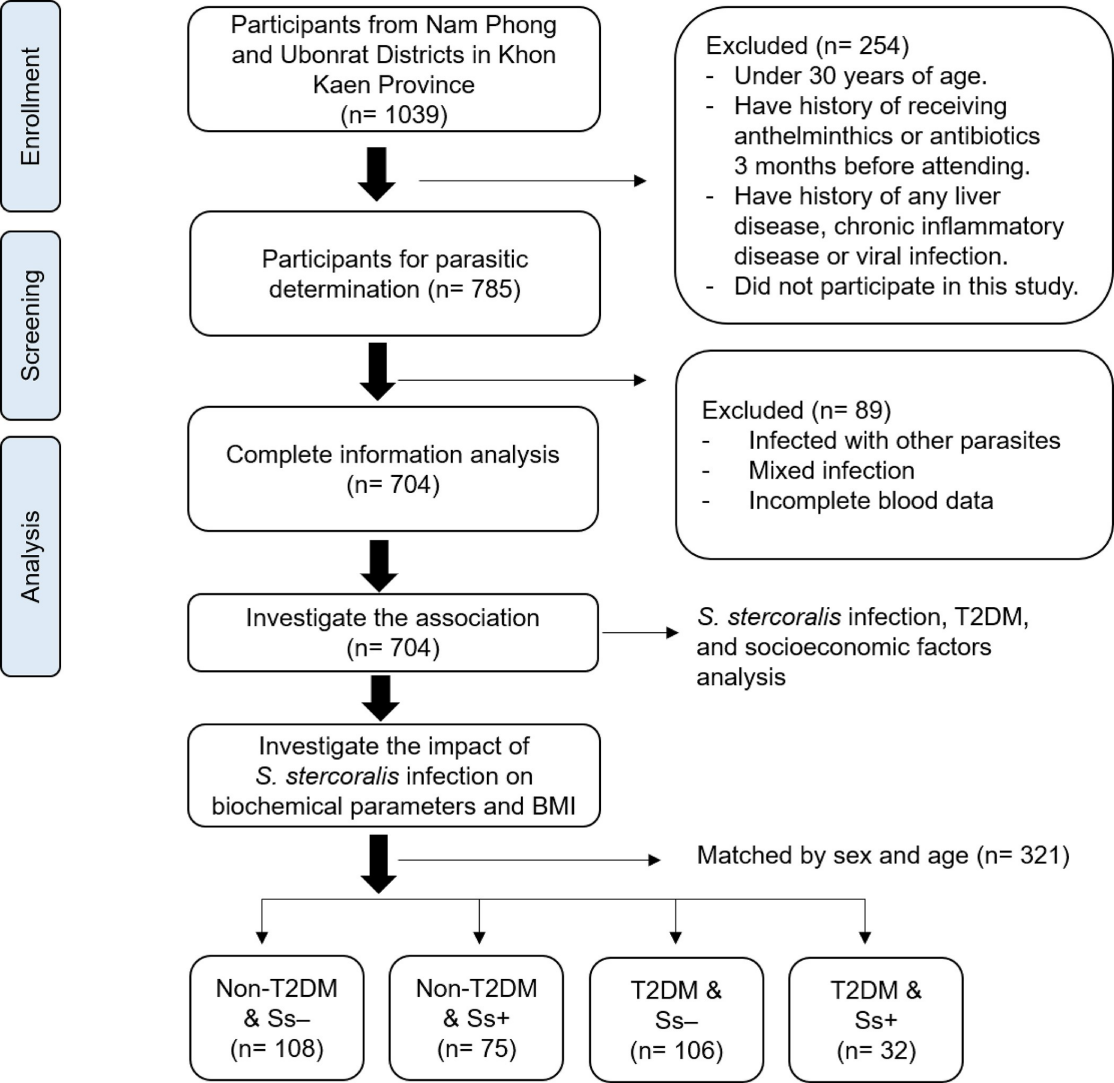
Flow diagram of the study participants and study design to investigate the association between *S*. *stercoralis* infection, T2DM and socioeconomic factors (n = 704), and their impact on BMI and kidney-related biochemical parameters (n = 321).

This study was a part of larger project, the Chronic Kidney Disease Prevention project in Northeast Thailand (CKDNET), Faculty of Medicine, Khon Kaen University. A cross-sectional study was conducted from October 2020 to May 2021 involving eight villages in two districts (Nam Phong and Ubonrat Districts) in Khon Kaen Province, northeastern Thailand (Fig 1). These locations were selected according to previous reports indicating a high prevalence of *S*. *stercoralis* infection [22, 23]. A total 1039 participants from these communities were enrolled to participate in this study. Of these, 785 individuals over 30 years of age, who had not received anthelminthics or antibiotics in the three months before attending the study, were included for screening of parasitic infections. Participants with a history of any chronic inflammatory disease or viral infection were excluded. Participants infected with other intestinal parasites (protozoa or helminths) and mixed infections (*S*. *stercoralis* with protozoa or other helminths) were also excluded. Only individuals singly infected with *S*. *stercoralis* based on detection using two parasitological methods (modified agar plate culture: mAPC, and the formalin-ethyl acetate concentration technique: FECT) were included (Fig 2).

To investigate the level of biochemical parameters related to kidney function of participants (with and without T2DM and/or *S*. *stercoralis* infection), blood and urine samples were collected. The estimated glomerular filtration rate (eGFR), urine albumin-to-creatinine ratio (UACR), serum creatinine, uric acid, alanine transaminase (ALT) and low-density lipoprotein cholesterol (LDL-C) were measured by the automated analyzer at the clinical laboratory of Srinagarind hospital, Khon Kaen University, and the body-mass index (BMI) of each participant was also determined. T2DM was diagnosed using fasting plasma-glucose (FPG) and/or glycated hemoglobin (HbA1c) levels. Participants were categorized into four groups for comparisons, namely, non-T2DM and not infected with *S*. *stercoralis* (Non-T2DM & Ss-), those with T2DM and not infected with *S*. *stercoralis* (T2DM & Ss-), non-T2DM and infected with *S*. *stercoralis* (Non-T2DM & Ss+) and those with T2DM and infected with *S*. *stercoralis* (T2DM & Ss+). Between groups, participants were randomly matched by sex and age according to the characteristics from the *S*. *stercoralis*-infected individuals (Fig 2).

### Sample size calculation

The sample size was calculated using a multiple logistic-regression formula [25] based on the desired primary outcome, that being an assessment of the association between diabetes and *S*. *stercoralis* infection. The proportions of *S*. *stercoralis*-infected individuals with and without T2DM (*P*_0_ = 0.161; *P*_1_ = 0.403) as previously reported [26] with 95% confidence interval (z = 1.96) and power at 80% (*β* = 0.20) were calculated. To reduce the multicollinearity, the variance inflation factor was adjusted (VIF = 0.7). The sample size was finally calculated as 506 individuals. However, a total of 785 subjects had returned the informed consent forms, stool, urine samples and allowed collection of blood samples, so we decided to include all 785 individuals.

### Stool sample collection and parasite identification

For collection of fresh stool samples, plastic containers were provided to the individual participants a few days before stool collection. Trained staff told all participants the procedure for collecting stool samples. Two parasitological methods (mAPC and FECT) were used to detect *S*. *stercoralis* infection and the presence of other intestinal parasites (e.g., protozoa and helminths). Preparation of samples and parasite detection were carried out as previously described [27, 28].

### Demographic characteristics and socioeconomic data collection and measurements

Demographic characteristics and socioeconomic status (age, gender, occupation, income, education level, level of alcohol intake, smoking and physical activity) were obtained by trained staff for each participant using a questionnaire. Weight, height, systolic and diastolic blood pressure of participants were directly measured in the community. Body-mass index (BMI) was calculated based on the body weight in kilograms divided by height in meters squared: two categories were recognized for data analysis based on the Asia-Pacific population BMI classification, <22.9 kg/m^2^ and ≥ 23 kg/m^2^ [29]. Systolic blood pressure of ≥140 mm Hg and diastolic ≥ 90 mm Hg were used as hypertension cut-off [30]. Smoking and alcohol intake histories were categorized as No or Yes and also as previous or current. Partaking of regular exercise until breaking a sweat was categorized as Yes or No.

### Collection of blood and urine samples and measurement of biochemical profiles

Blood and urine samples were collected in the morning on the same day that participants returned the informed consent forms and fresh stool samples. Participants were required to fast for at least 12 hr before the blood collection day. Fasting plasma-glucose (FPG) and/or glycated-hemoglobin (HbA1c) levels were used to determine T2DM status. Individuals with glycated hemoglobin (HbA1c) ≥6.5% (according to WHO guidelines) and/or fasting plasma glucose (FPG) ≥7.0 mmol/L or ≥126 mg/dL (based on the American Diabetic Association recommendation) were regarded as having T2DM [31]. Levels of direct low-density lipoprotein cholesterol (LDL-C) ≤129 mg/dL were defined as normal. The eGFR was classified into five levels: normal (eGFR ≥90 mL/min/1.73 m^2^); kidney function severity, mild (eGFR 60–89 mL/min/1.73 m^2^), moderate (eGFR 30–59 mL/min/1.73 m^2^), severe (eGFR 15–29 mL/min/1.73 m^2^), and kidney failure (eGFR <15 mL/min/1.73 m^2^) [32]. Levels of serum creatinine ranging from 0.67–1.17 mg/dL, uric acid (3.5–8.7 mg/dL), and ALT (0–33 U/L) were defined as normal. A urine albumin-to-creatinine ratio from urine analysis was defined as normal (<30 mg/g), moderately increased risk (30–300 mg/g), and greatly increased risk of kidney disease (>300 mg/g) [32].

### Statistical analyses

Demographic characteristics, socioeconomic status and parasite infections were described as mean, standard deviation (SD), number of participants and percentages. A multiple logistic regression was used to analyze the association factors. To fit the multivariable model, p*-*values ≤ 0.25 of variable factors were initially selected using backward elimination and Hosmer-Lemeshow goodness-of-fit tests. Adjusted (sex, age, smoking, alcohol intake, BMI, and exercise) factors were then calculated. *P* values <0.05 were considered to be statistically significant and adjusted odds ratio (adjusted OR) with 95% confidence intervals (95% CI) were presented. Comparisons of mean differences of biochemical parameters (LDL-C, eGFR, serum creatinine, UACR, uric acid and ALT) and BMI between T2DM and non-T2DM groups with and without *S*. *stercoralis* infection were employed using one-way-ANOVA with Bonferroni post-hoc tests. These analyses were performed using STATA v10.1 (Stata Corporation, College Station, TX, USA).

## Results

### Study population and characteristics

In total, 785 participants who met the inclusion criteria were enrolled. Individuals with other intestinal parasite infections (helminths, protozoans and/or mixed infections) other than strongyloidiasis, or for whom there were missing data, were excluded as shown in S1 Table. Data from 704 individuals, who had completed and signed the informed-consent forms, completed the questionnaire, provided blood and urine samples and either had a mono-infection with *S*. *stercoralis* or were free of all parasites, were finally analyzed.

Most participants in this study were females (69.46%), age 58.23±9.38 years old, main occupation stated as agriculture (72.44%) and had not completed primary school education (72.44%) (Table 1). One hundred and seven people (15.20%) were infected with *S*. *stercoralis* and 283 people (40.20%) were diagnosed with T2DM. Thirty-two individuals (32/283; 11.31%) with T2DM were infected with *S*. *stercoralis*, while non-T2DM participants were infected in 75/421 (17.82%) cases.

**Table 1:** Demographic characteristics and socioeconomic status of the population (*n* = 704).

Variables	n or mean ± SD	Percentage (%)
**Gender**		
Female	489	69.46
Male	215	30.54
**Age**		
30–39	22	3.13
40–49	98	13.92
50–59	273	38.78
60–69	225	31.96
>70	86	12.22
Mean ± SD	58.23±9.38	
**Weight (kg)**	60.64±9.83	
**Height (cm)**	157.19±7.19	
**Education level**		
Primary school or lower	510	72.44
Higher than primary school	194	27.56
**Main occupation**		
Un-employed	91	12.93
Un-agriculturist	103	14.63
Agriculturist	510	72.44
**Income per month (baht)**		
< 10,000	639	90.77
> 10,000	65	9.23
**BMI (kg/m** ^**2**^ **)**	24.55±3.71	
**Blood pressure of systolic**	126.85±16.56	
**Blood pressure of diastolic**	82.06±10.42	
**Smoking**		
No	583	82.81
Yes, previous	66	9.38
Yes, current	55	7.81
**Alcohol intake**		
No	416	59.09
Yes, previous	115	16.34
Yes, current	173	24.57
**Exercise**		
No	209	29.69
Yes (until breaking a sweat)	495	70.31
**T2DM status**		
No	421	59.80
Yes	283	40.20

### Association between *S*. *stercoralis* infection, T2DM and socioeconomic factors

Univariable analysis (crude analysis) indicated that the factors including being male, aged over 60 years, with occupational exposure (agriculturist), failing to complete primary school and having a history of alcohol intake, were significantly associated with *S*. *stercoralis* infection (*p* < 0.05) (Table 2). After adjusting for these factors, multivariable logistic regression analysis results are shown in Table 3. The factors (sex, age, education level, and alcohol intake history) remained significantly associated with *S*. *stercoralis* infection (*p* < 0.05). T2DM status was significantly negatively associated with *S*. *stercoralis* infection (Adjusted OR  =  0.49; 95% CI: 0.30, 0.78; *p* = 0.003).

**Table 2:** Univariable logistic regression analyses of the association between demographic characteristics, socioeconomic factors and *S*. *stercoralis* infection (*n =* 704).

Variables	*S*. *stercoralis*		Crude OR	95%CI	*p*-value
	Yes	No			
	n	n			
**Gender**					
Female	47	442	1		
Male	60	155	3.640	2.384, 5.559	<0.001
**Age**					
30–59	44	349	1		
≥60	63	248	2.015	1.326, 3.060	0.001
**Education level**					
Higher than primary school	14	153	1		
Primary school or lower	93	444	2.289	1.267, 4.134	0.006
**Main occupation**					
Un-employed	11	80	1.150	0.473, 2.794	0.758
Un-agriculturist	11	92	1		
Agriculturist	85	425	1.672	0.858, 3.259	0.131
**Income per month (baht)**					
< 10,000	100	539	1		
> 10,000	7	58	0.651	0.289, 14.466	0.300
BMI (kg/m^2^)					
<22.9	66	336	1		
≥23	41	261	0.799	0.511, 1.243	0.298
**Smoking**					
No	71	512	1		
Yes, previous	15	51	2.121	1.133, 3.970	0.019
Yes, current	21	34	4.454	2.449, 8.098	<0.001
**Alcohol intake**					
No	44	373	1		
Yes, previous	22	93	2.000	1.142, 3.501	0.015
Yes, current	41	132	2.626	1.642, 4.199	<0.001
**Exercise**					
No	29	180	1		
Yes (until breaking a sweat)	78	417	1.161	0.732, 1.840	0.525
**T2DM status**					
No	75	346	1		
Yes	32	251	0.588	0.377, 0.917	0.019

**Table 3:** Multivariable logistic regression analyses of factors associated with *S*. *stercoralis* infection (*n =* 704).

Variables	*S*. *stercoralis*		Crude OR	Adjusted OR	95% CI	*p*-value
	Yes	No				
	n	n				
**Sex**						
Female	47	442	1	1		
Male	60	155	3.640	2.936	1.759, 4.896	<0.001
**Education level**						
Higher than primary school	14	153	1	1		
Lower than primary school	93	444	2.289	2.062	1.069, 3.979	0.031
**Age**						
30–59	44	349	1	1		
≥60	63	248	2.015	1.618	1.017, 2.572	0.042
**Alcohol intake**						
No	44	373	1	1		
Yes, previous	22	93	2.000	1.146	0.604, 2.171	0.676
Yes, current	41	132	2.626	1.986	1.135, 3.477	0.016
**T2DM status**						
No	75	346	1	1		
Yes	32	251	0.588	0.485	0.303, 0.777	0.003

### Impact of *S*. *stercoralis* infection on diabetic complication-related renal biochemical parameters

**Fig 3:**
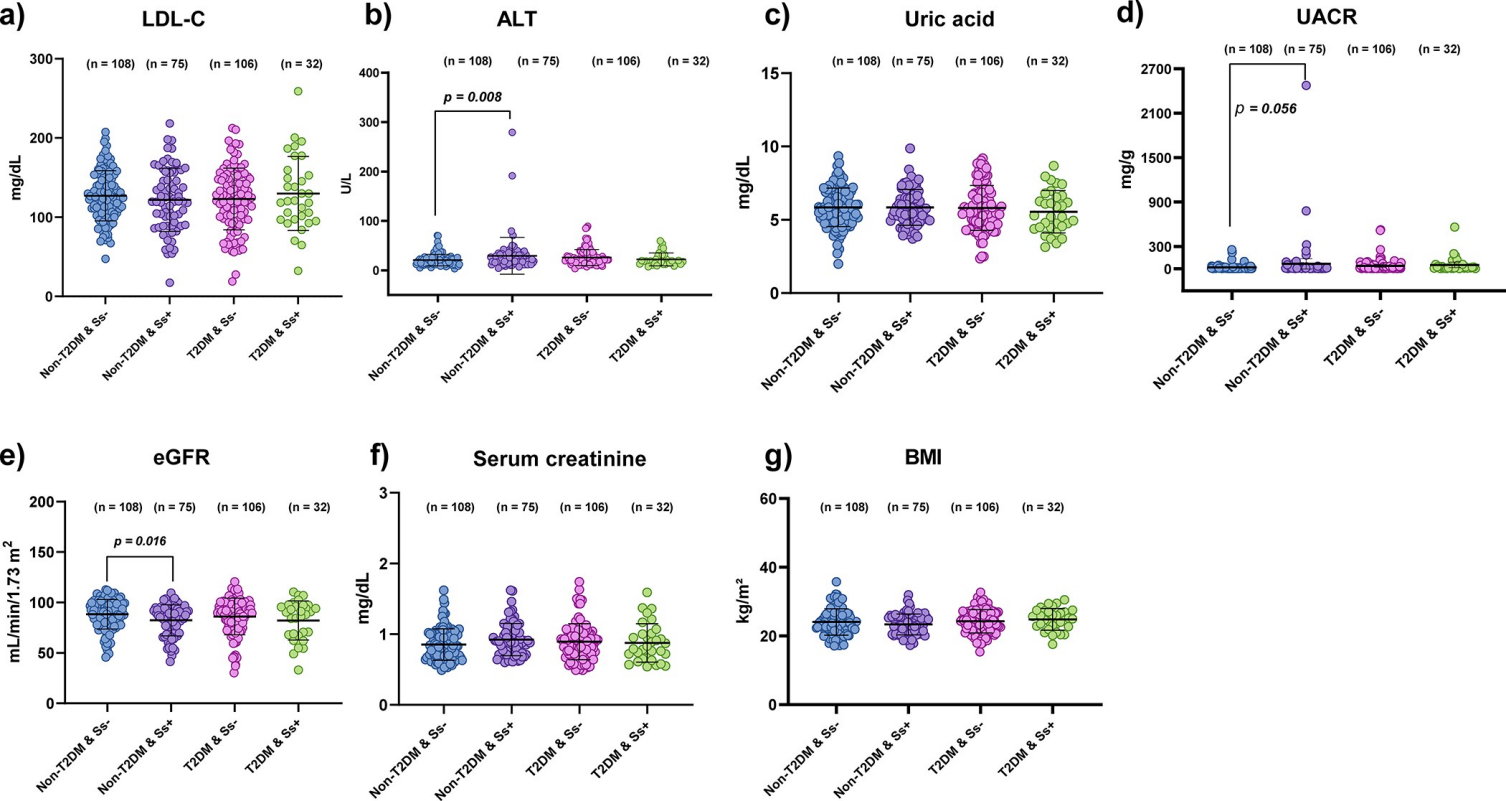
Association of *S*. *stercoralis* infection with biochemical profiles BMI and T2DM (matching sex and age between groups). a): Direct low-density lipoprotein cholesterol: LDL-C; b): Alanine transaminase: ALT; c): Uric acid; d): Urine albumin-to-creatinine ratio: UACR; e): Estimated glomerular filtration rate: eGFR; f): Serum creatinine; g): Body mass index: BMI. Lines indicate mean and standard deviation (SD) of data. Statistical significance based on one-way ANOVA.

The effect of *S*. *stercoralis* infection on biochemical parameters related to kidney function and BMI in T2DM and non-T2DM groups is shown in Fig 3. There were no statistically significant differences in sex, age, level of LDL-C, uric acid, serum creatinine and BMI between the four groups (*p* > 0.05) (S2 Table). However, the eGFR level in the Non-T2DM & Ss+ group was significantly lower than in the Non-T2DM & Ss- group (Mean difference: -6.05; *p* = 0.016; 95%CI: -10.95, -1.15), and the ALT level was significantly higher than in the Non-T2DM & Ss- group (Mean difference: 8.87; *p* = 0.008; 95%CI: 2.38, 15.35). The UACR level in the Non-T2DM & Ss+ group was also higher than in the Non-T2DM & Ss- group (mean difference: 47.54; *p* = 0.056; 95%CI: -1.13, 96.21) (S3 Table).

## Discussion

In this study, we investigated the association between *S*. *stercoralis* infection and T2DM in Khon Kaen Province, Thailand. Overall, 15.20% of participants were singly infected with *S*. *stercoralis* (11.31% among those with T2DM and 17.82% among those without T2DM). Certain demographic and socioeconomic factors (being male, being older, having education level lower than primary school and a history of alcohol intake) were significantly associated with *S*. *stercoralis* infection risk, similar to previous findings in Thailand [22, 23] and other countries [33, 34]. These same demographic and socioeconomic factors are commonly known to predispose people to infection and to reduce the efficiency of the immune response [35]. After adjusting for these factors, T2DM exhibited an inverse association with *S*. *stercoralis* infection. Similar findings have been reported from Australia [7], India [36], and in studies on other soil-transmitted helminths in Indonesia and other helminths in rural China and Ethiopia [6, 8, 37]. It has been postulated that nutrition, gut homeostasis and immunoregulatory response during parasite infection are responsible for this relationship [10, 36, 38]. Helminth infection can alter Th1/Th2 immune polarization, reduce systemic levels of Th1/Th17 pro-inflammatory cytokines (IL-4 over IL-17, TNF-α and IFN-γ), switch macrophages from M1 to M2 pattern, or change intestinal microbial diversity, all of which lead to increased insulin sensitivity [6, 39, 40]. In contrast, prior study on hospital-based subjects has reported a high frequency of *S*. *stercoralis* infection in T2DM patients using serology method [41], which may differently discovery from the community-based study, diagnosis method uses, and host immunity. However, a previous cohort study in T2DM patients from a clinic in remote community in Australia followed up 3 years after treatment of positive IgG *S*. *stercoralis* cases showed that treated subjects were more likely to be newly diagnosed with T2DM than those who were not treated [42]. Eliminating of *S*. *stercoralis* infection by treatment might leave the patients more susceptible to developing T2DM. However, serological methods for measuring positive IgG, such as ELISA, can be ineffective in distinguishing between active and historical *S*. *stercoralis* infection [15, 43]. Although exposure to the seropositive *S*. *stercoralis* seems to protect T2DM-driven inflammation, it does not indicate the presence of an active parasite infection, which may be less likely to provide the immune regulation against T2DM when compared to an active parasite infection [13]. Long-term investigation of active *S*. *stercoralis* infection may be of interest.

Several studies have indicated that lipid profiles and metabolic outcomes in both T2DM subjects and animal models can be altered by helminth infections (*Schistosoma* spp, *Opisthorchis viverrini* and filaria) or parasite-derived antigens [37, 44–46]. In this study, we have presented data on biochemical parameters relating to kidney function in individuals infected with an intestinal helminth, *S*. *stercoralis*. Participants were matched by sex and age. We did not find any significant differences in the levels of LDL-C, serum biochemical parameters (serum creatinine and uric acid) and BMI in both T2DM and non-T2DM with or without *S*. *stercoralis* infection (Fig 3). These findings are contrary to previous work conducted in schistosomiasis [8, 19, 37], opisthorchiasis [47] and soil-transmitted helminths [48], which have all reported that infection with parasites could lead to changed serum lipid levels (LDL-C and/or HDL levels,) or BMI, which in turn helps to prevent the development of disease complications [21, 49]. These differences might be a consequence of differing participant age groups, recently of infection, worm burdens, geographical area, culture, and dietary habits. Moreover, unlike *S*. *stercoralis*, *Schistosoma* species live in blood vessels and tissues, thus possibly affecting blood and lipid profiles more directly than do intestinal helminths [50] and suggesting that outcomes might depend on the type of parasite and its site in the host, intensity of infection, host gut microbial diversity and host immunity.

Interestingly, we found a low level of eGFR in both *S*. *stercoralis*-infected groups (with or without T2DM), which was significantly lower in the non-T2DM *S*. *stercoralis*-infected group. The same group also had high levels of ALT and UACR compared to the non-T2DM uninfected group. This observation indicated the low level of biochemical parameters related to kidney function of host during the infection of *S*. *stercoralis* in both T2DM and without T2DM. We suggest that infection with *S*. *stercoralis* might be a risk factor for increased morbidity in the human host. This might be attributed to the effects of *S*. *stercoralis* infection on organ systems associated with kidney disease risk. Glomerular pathology might be related to immune-complex deposition of *S*. *stercoralis* antigens in the kidneys [51, 52]. Infection with *S*. *stercoralis* might also changes in gut microbial diversity of the host, leading to production of some uremic toxins that reach the kidneys. However, further study of relevant molecular pathways is required.

This study has some limitations. Firstly, it is a cross-sectional study that cannot assign the causal effect. Our observations only reflect a moment in time in the interaction between *S*. *stercoralis* infection and T2DM as well as their biochemical parameters related to kidney function. Although we found an inverse correlation of *S*. *stercoralis* infection with T2DM, other differences, such as lifestyle, dietary habits or preventive practices toward parasite infection between T2DM and non-T2DM patients have yet to be identified. Secondly, despite higher UACR levels in the non-T2DM infected group, they were still not statistically significant due to the small sample size. Increasing the sample size will be beneficial. Thirdly, we could say nothing about the duration of *S*. *stercoralis* infection in our subjects. Participants in this study seemed to have chronic strongyloidiasis, which is characterized by a low intensity of worms. As a result, the impacts of *S*. *stercoralis* infection were unable to provide information regarding alterations that may occur during the early stages of infection. We also did not count the number of *S*. *stercoralis* larvae due to the low sensitivity of the FECT method. Stool samples were collected one time only from each participant, so any count that we did would likely be an underestimate in cases of chronic infection. Indeed, we may have failed to identify some infected individuals, perhaps leading to inappropriate group assignment. However, we also used the modified APC technique to increase sensitivity of larvae detection based on cultivation. This technique was also performed in the community and used fresh stool sample in combination with FECT to reduce the sample selection bias for group classification. In future work, the fecal samples should be collected over multiple consecutive days and examined using molecular and/or immunological techniques to confirm infection status of participants and whether current or past infection [53]. Counting the number of worms, identifying acute or chronic infection, and increasing sample sizes are necessary. Investigation of the long-term effects of the use of ivermectin or other anthelminthics on biochemical parameters both before and after treatment for *S*. *stercoralis* will also be of value.

## Conclusion

This study is the first to demonstrate the relationship between infection with the intestinal helminth *S*. *stercoralis* and T2DM in communities of northeastern Thailand. Prevalence of *S*. *stercoralis* infection was inversely correlated with T2DM status, but individuals infected with this parasite showed low eGFR levels and higher ALT and UACR levels than uninfected individuals. Infection with *S*. *stercoralis* might be a potential risk for kidney disease, judging from the relevant biochemical parameters in both T2DM and non-T2DM subjects: this requires further study.

## Supporting information

S1 Table Frequency of intestinal parasitic infections using FECT and mAPC (n = 785). (DOCX)

S2 Table Characteristics of participants from four groups (n = 321). (DOCX)

S3 Table Analysis the effect of *S*. *stercoralis* infection on biochemical parameters in T2DM and non-T2DM participants based on one-way ANOVA (n = 321). (DOCX)
