# Correction: Association of Strongyloides stercoralis infection and type 2 diabetes mellitus in northeastern Thailand: Impact on diabetic complication-related renal biochemical parameters

**DOI:** 10.1371/journal.pone.0303774

**Published:** 2024-05-09

**Authors:** Manachai Yingklang, Apisit Chaidee, Rungtiwa Dangtakot, Chanakan Jantawong, Ornuma Haonon, Chutima Sitthirach, Nguyen Thi Hai, Ubon Cha’on, Sirirat Anutrakulchai, Supot Kamsa-ard, Somchai Pinlaor

In the Human ethical statement subsection of Materials and methods, there is an error in first sentence. The correct sentence is: This study was approved by the human ethical review committee of Khon Kaen University, Thailand (HE631673) following the principles of the Declaration of Helsinki.
