# Supplementary material for: Association of Strongyloides stercoralis infection and type 2 diabetes mellitus in northeastern Thailand: Impact on diabetic complication-related renal biochemical parameters
Source: PLoS One. 2022 May 31;17(5):e0269080. doi: 10.1371/journal.pone.0269080 (PMC9154194; doi:10.1371/journal.pone.0269080)
Supplement: S1 Table — (DOCX) [file pone.0269080.s001.docx]

**S1 Table**

Frequency of intestinal parasitic infections using FECT and mAPC (*n*=785)

| **Type of parasitic infections** | **n** |
| --- | --- |
| **All** | 152 |
| **Helminths (mono-infection)** |  |
| *Strongyloides stercoralis* | 107 |
| *Opisthorchis viverrini* | 2 |
| *Minute intestinal fluke* | 1 |
| **Protozoa (mono-infection)** |  |
| *Blastocystis* *hominis* | 15 |
| *Sarcocystis* sp. | 10 |
| *Giardia lamblia* | 3 |
| *Entamoeba coli* | 1 |
| **Mixed infections** |  |
| *Strongyloides stercoralis +Sarcocystis* sp. | 3 |
| *Strongyloides stercoralis* + Hookworm | 2 |
| *Strongyloides stercoralis + Blastocystis* *hominis* | 2 |
| *Strongyloides stercoralis* + *Opisthorchis viverrini* | 1 |
| *Sarcocystis* sp. *+ Taenia* sp. | 1 |
