# Supplementary material for: Association of Strongyloides stercoralis infection and type 2 diabetes mellitus in northeastern Thailand: Impact on diabetic complication-related renal biochemical parameters
Source: PLoS One. 2022 May 31;17(5):e0269080. doi: 10.1371/journal.pone.0269080 (PMC9154194; doi:10.1371/journal.pone.0269080)
Supplement: S2 Table — (DOCX) [file pone.0269080.s002.docx]

**S2 Table**

Characteristics of participants from four groups (*n=*321).

| **Variables** | **Infected with *S. stercoralis*** | | **Un-infected with *S. stercoralis*** | | ***p*-value** |
| --- | --- | --- | --- | --- | --- |
|  | **non-T2DM**  **(n = 75)** | **T2DM**  **(n = 32)** | **Non-T2DM**  **(n = 108)** | **T2DM**  **(n = 106)** |  |
| **Gender** | | | | | |
| Female | 28 | 19 | 45 | 34 | 0.225 |
| Male | 47 | 13 | 63 | 72 |  |
| **Age** | | | | | |
| Mean ±SD | 60.88±8.15 | 61.5 ±7.40 | 60.15 ±7.98 | 61.73 ±9.24 | 0.309 |

| **Variables** | **Mean ± SD** |
| --- | --- |
| **LDL-C (<129 mg/dL: normal)** |  |
| No-SS_infection & non-T2DM | 126.94 ± 31.67 |
| No-SS_infection & T2DM | 122.92 ± 38.97 |
| SS_infection & non-T2DM | 121.93 ± 39.70 |
| SS_infection & T2DM | 129.85 ± 46.74 |
| **ALT (0-33 U/L: normal)** |  |
| No-SS_infection & non-T2DM | 20.73 ± 11.88 |
| No-SS_infection & T2DM | 25.79 ± 16.38 |
| SS_infection & non-T2DM | 29.59 ± 37.46 |
| SS_infection & T2DM | 22.02 ± 13.18 |
| **Uric acid (>3.5-8.7 mg/dL: normal)** |  |
| No-SS_infection & non-T2DM | 5.85 ± 1.32 |
| No-SS_infection & T2DM | 5.81 ± 1.54 |
| SS_infection & non-T2DM | 5.85 ± 1.22 |
| SS_infection & T2DM | 5.55 ± 1.44 |
| **eGFR (>90: normal, 60-89: mild)** |  |
| No-SS_infection & non-T2DM | 88.38 ± 14.69 |
| No-SS_infection & T2DM | 86.09 ± 18.19 |
| SS_infection & non-T2DM | 82.34 ± 15.43 |
| SS_infection & T2DM | 82.14 ± 19.42 |
| **Serum creatinine (>0.67- 1.17 mg/dL: normal)** |  |
| No-SS_infection & non-T2DM | 0.853 ± 0.22 |
| No-SS_infection & T2DM | 0.892 ± 0.25 |
| SS_infection & non-T2DM | 0.924 ± 0.22 |
| SS_infection & T2DM | 0.875 ± 0.27 |
| **BMI** |  |
| No-SS_infection & non-T2DM | 24.05 ± 3.85 |
| No-SS_infection & T2DM | 24.27 ± 3.40 |
| SS_infection & non-T2DM | 23.35 ± 3.01 |
| SS_infection & T2DM | 24.77 ± 3.12 |
| **UACR** |  |
| No-SS_infection & non-T2DM | 16.44± 40.89 |
| No-SS_infection & T2DM | 38.92± 117.17 |
| SS_infection & non-T2DM | 63.97± 299.08 |
| SS_infection & T2DM | 49.93± 105.72 |
