# Supplementary material for: Association of Strongyloides stercoralis infection and type 2 diabetes mellitus in northeastern Thailand: Impact on diabetic complication-related renal biochemical parameters
Source: PLoS One. 2022 May 31;17(5):e0269080. doi: 10.1371/journal.pone.0269080 (PMC9154194; doi:10.1371/journal.pone.0269080)
Supplement: S3 Table — (DOCX) [file pone.0269080.s003.docx]

**S3 Table**

Analysis the effect of *S. stercoralis* infection on biochemical parameters in T2DM and non-T2DM participants **based on** One-way ANOVA (*n=*321).

| **Variables** | **Mean Different** | **SE** | **t** | **P>\|t\|** | **95% CI (Lower, Upper)** |
| --- | --- | --- | --- | --- | --- |
| **1. ALT** |  |  |  |  |  |
| (no-SS_infection & T2DM) vs (no-SS_infection & non-T2DM) | 5.064 | 2.999 | 1.69 | 0.092 | -0.836, 10.965 |
| (SS_infection & non-T2DM) vs (no-SS_infection & non-T2DM) | 8.866 | 3.297 | 2.69 | 0.008 | 2.379, 15.353 |
| (SS_infection & T2DM) vs (no-SS_infection & non-T2DM) | 1.295 | 4.415 | 0.29 | 0.769 | -7.391, 9.981 |
| (SS_infection & non-T2DM) vs (no-SS_infection & T2DM) | 3.801 | 3.310 | 1.15 | 0.252 | -2.710, 10.314 |
| (SS_infection & T2DM) vs (no-SS_infection & T2DM) | -3.769 | 4.424 | -0.85 | 0.395 | -12.475, 4.935 |
| (SS_infection & T2DM) vs (SS_infection & non-T2DM) | -7.571 | 4.631 | -1.63 | 0.103 | -16.684, 1.541 |
| **2. LDL-C** |  |  |  |  |  |
| (no-SS_infection & T2DM) vs (no-SS_infection & non-T2DM) | -4.019 | 5.158 | -0.78 | 0.436 | -14.168, 6.128 |
| (SS_infection & non-T2DM) vs (no-SS_infection & non-T2DM) | -5.007 | 5.670 | -0.88 | 0.378 | -16.164, 6.149 |
| (SS_infection & T2DM) vs (no-SS_infection & non-T2DM) | 2.915 | 7.593 | 0.38 | 0.701 | -12.024, 17.855 |
| (SS_infection & non-T2DM) vs (no-SS_infection & T2DM) | -.987 | 5.692 | -0.17 | 0.862 | -12.187, 10.212 |
| (SS_infection & T2DM) vs (no-SS_infection & T2DM) | 6.935 | 7.609 | 0.91 | 0.363 | -8.036, 21.907 |
| (SS_infection & T2DM) vs (SS_infection & non-T2DM) | 7.922 | 7.966 | 0.99 | 0.321 | -7.750, 23.596 |
| **3. eGFR** |  |  |  |  |  |
| (no-SS_infection & T2DM) vs (no-SS_infection & non-T2DM) | -2.297 | 2.267 | -1.01 | 0.312 | -6.758, 2.164 |
| (SS_infection & non-T2DM) vs (no-SS_infection & non-T2DM) | -6.045 | 2.492 | -2.43 | 0.016 | -10.950, -1.149 |
| (SS_infection & T2DM) vs (no-SS_infection & non-T2DM) | -6.242 | 3.338 | -1.87 | 0.062 | -12.809, 0.325 |
| (SS_infection & non-T2DM) vs (no-SS_infection & T2DM) | -3.748 | 2.502 | -1.50 | 0.135 | -8.672, 1.175 |
| (SS_infection & T2DM) vs (no-SS_infection & T2DM) | -3.944 | 3.345 | -1.18 | 0.239 | -10.526, 2.637 |
| (SS_infection & T2DM) vs (SS_infection & non-T2DM) | -0.196 | 3.502 | -0.06 | 0.955 | -7.086, 6.694 |
| **4. Uric acid** | **Mean Different** | **SE** | **t** | **P>\|t\|** | **95% CI (Lower, Upper)** |
| (no-SS_infection & T2DM) vs (no-SS_infection & non-T2DM) | -0.035 | 0.189 | -0.19 | 0.853 | -0.407, 0.337 |
| (SS_infection & non-T2DM) vs (no-SS_infection & non-T2DM) | 0.001 | 0.208 | 0.01 | 0.993 | -0.407, 0.411 |
| (SS_infection & T2DM) vs (no-SS_infection & non-T2DM) | -0.296 | 0.278 | -1.07 | 0.287 | -0.844, 0.251 |
| (SS_infection & non-T2DM) vs (no-SS_infection & T2DM) | 0.037 | 0.208 | 0.18 | 0.859 | -0.373, 0.447 |
| (SS_infection & T2DM) vs (no-SS_infection & T2DM) | -0.261 | 0.279 | -0.94 | 0.349 | -0.811, 0.287 |
| (SS_infection & T2DM) vs (SS_infection & non-T2DM) | -0.298 | 0.292 | -1.02 | 0.307 | -0.873, 0.276 |
| **5. Serum creatinine** |  |  |  |  |  |
| (no-SS_infection & T2DM) vs (no-SS_infection & non-T2DM) | 0.040 | 0.032 | 1.23 | 0.221 | -0.024, 0.105 |
| (SS_infection & non-T2DM) vs (no-SS_infection & non-T2DM) | 0.0706 | 0.036 | 1.95 | 0.052 | -0.001, 0.141 |
| (SS_infection & T2DM) vs (no-SS_infection & non-T2DM) | 0.022 | 0.048 | 0.46 | 0.645 | -0.072, 0.117 |
| (SS_infection & non-T2DM) vs (no-SS_infection & T2DM) | 0.030 | 0.036 | 0.83 | 0.405 | -0.041, 0.101 |
| (SS_infection & T2DM) vs (no-SS_infection & T2DM) | -0.018 | 0.048 | -0.37 | 0.711 | -0.113, 0.077 |
| (SS_infection & T2DM) vs (SS_infection & non-T2DM) | -0.048 | 0.050 | -0.95 | 0.342 | -0.148, 0.051 |
| **6. BMI** |  |  |  |  |  |
| (no-SS_infection & T2DM) vs (no-SS_infection & non-T2DM) | 0.220 | 0.472 | 0.47 | 0.641 | -0.708, 1.149 |
| (SS_infection & non-T2DM) vs (no-SS_infection & non-T2DM) | -0.703 | 0.519 | -1.36 | 0.176 | -1.725, 0.317 |
| (SS_infection & T2DM) vs (no-SS_infection & non-T2DM) | 0.717 | 0.695 | 1.03 | 0.303 | -0.650, 2.085 |
| (SS_infection & non-T2DM) vs (no-SS_infection & T2DM) | -0.924 | 0.521 | -1.77 | 0.077 | -1.949, 0.100 |
| (SS_infection & T2DM) vs (no-SS_infection & T2DM) | 0.496 | 0.696 | 0.71 | 0.476 | -0.873, 1.867 |
| (SS_infection & T2DM) vs (SS_infection & non-T2DM) | 1.421 | 0.729 | 1.95 | 0.052 | -0.013, 2.856 |

| **7. UACR** | **Mean Different** | **SE** | **t** | **P>\|t\|** | **95% CI (Lower, Upper)** |
| --- | --- | --- | --- | --- | --- |
| (no-SS_infection & T2DM) vs (no-SS_infection & non-T2DM) | 22.485 | 22.501 | 1.00 | 0.318 | -21.786, 66.757 |
| (SS_infection & non-T2DM) vs (no-SS_infection & non-T2DM) | 47.542 | 24.737 | 1.92 | **0.056** | -1.128, 96.213 |
| (SS_infection & T2DM) vs (no-SS_infection & non-T2DM) | 33.488 | 33.124 | 1.01 | 0.313 | -31.683, 98.660 |
| (SS_infection & non-T2DM) vs (no-SS_infection & T2DM) | 25.057 | 24.833 | 1.01 | **0.314** | -23.801, 73.915 |
| (SS_infection & T2DM) vs (no-SS_infection & T2DM) | 11.003 | 33.196 | 0.33 | 0.741 | -54.309, 76.316 |
| (SS_infection & T2DM) vs (SS_infection & non-T2DM) | -14.053 | 34.750 | -0.40 | 0.686 | -82.424, 54.317 |
